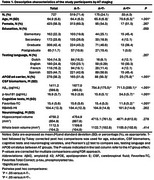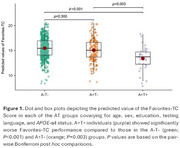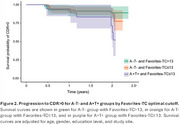# Detecting early memory changes in preclinical Alzheimer's disease using TabCAT Favorites Test: data from the European Prevention of Alzheimer's Dementia cohort

**DOI:** 10.1002/alz70856_104291

**Published:** 2025-12-26

**Authors:** Anna Brugulat‐Serrat, Elena Tsoy, Gonzalo Sánchez‐Benavides, Marta Milà‐Alomà, Oriol Grau‐Rivera, Leslie S. Gaynor, Juan Domingo Gispert, Joel H Kramer, Katherine L. Possin, for the European Prevention of Alzheimer's Dementia (EPAD) Consortium

**Affiliations:** ^1^ Faculty of Medicine, University of Vic‐Central University of Catalonia (UVic‐UCC), Vic/Manresa, Catalonia, Spain; ^2^ Global Brain Health Institute, San Francisco, CA, USA; ^3^ IMIM (Hospital del Mar Medical Research Institute), Barcelona, Spain; ^4^ Barcelonaβeta Brain Research Center (BBRC), Pasqual Maragall Foundation, Barcelona, Spain; ^5^ Centro de Investigación Biomédica en Red de Fragilidad y Envejecimiento Saludable (CIBERFES), Instituto de Salud Carlos III, Barcelona, Spain; ^6^ Memory and Aging Center, University of California San Francisco, San Francisco, CA, USA; ^7^ Global Brain Health Institute, University of California, San Francisco, San Francisco, CA, USA; ^8^ Barcelonaβeta Brain Research Center (BBRC), Barcelona, Spain; ^9^ Centro de Investigación Biomédica en Red de Fragilidad y Envejecimiento Saludable (CIBERFES), 28089, Madrid, Spain; ^10^ Department of Veterans Affairs Medical Center, Northern California Institute for Research and Education (NCIRE), San Francisco, CA, USA; ^11^ Centro de Investigación Biomédica en Red de Fragilidad y Envejecimiento Saludable (CIBERFES), Instituto de Salud Carlos III, Madrid, Spain; ^12^ Servei de Neurologia, Hospital del Mar, Barcelona, Spain; ^13^ Vanderbilt University Medical Center, Nashville, TN, USA; ^14^ Centro de Investigación Biomédica en Red Bioingeniería, Biomateriales y Nanomedicina (CIBER‐BBN), Instituto de Salud Carlos III, Madrid, Spain; ^15^ Memory and Aging Center, UCSF Weill Institute for Neurosciences, University of California, San Francisco, San Francisco, CA, USA; ^16^ Global Brain Health Institute, University of California San Francisco, San Francisco, CA, USA; ^17^ Memory and Aging Center, Weill Institute for Neurosciences, University of California, San Francisco, San Francisco, CA, USA; ^18^ Global Brain Health Institute (GBHI), University of California San Francisco (UCSF); & Trinity College Dublin, San Francisco, CA, USA

## Abstract

**Background:**

Sensitive memory paradigms are needed to detect subtle memory changes associated with early Alzheimer's disease (AD) pathology in individuals without established clinical symptomatology. We explored cross‐sectional associations between performance on a brief computerized episodic memory test with AD cerebrospinal fluid (CSF) biomarker status and future clinical progression in a large multinational sample of cognitively unimpaired (CU) individuals from the EPAD Longitudinal Cohort Study (LCS).

**Method:**

727 CU individuals from the EPAD LCS with valid TabCAT Favorites memory performance and CSF biomarkers were included. Episodic memory was also evaluated using the RBANS Memory Index (RBANS‐MI). Independent multinomial logistic regression models were performed for each memory measure to examine the associations between episodic memory performance and AT stages. Covariates included age, sex, education level, testing language, and *APOE*‐ε4 status. The optimal Favorites cutoff maximizing Youden index was derived for the discriminative analysis. A Cox proportional hazard model was used to examine how the optimal cutoff within AT groups predicts change in Clinical Dementia Rating (CDR) scores.

**Result:**

Descriptive characteristics of the participants are shown in Table 1. Compared to A‐T‐ individuals, poorer Favorites cross‐sectional performance was associated with an increased likelihood of A+T+ status (OR=0.92, 95% CI=0.86‐0.99, *p* = .030), but not A+T‐ status (OR=1.0, 95% CI=0.97‐1.04, *p* = .813, Figure 1). There were no significant associations between AT status and RBANS‐MI. Favorites showed a significant discriminative validity in predicting progression to CDR>0 only among A+T+ individuals (*p* = 0.014, Figure 2).

**Conclusion:**

Our findings showed that the tablet‐based TabCAT Favorites is a sensitive measure for detecting cognitive changes in earlier stages of the AD continuum, representing a valuable alternative to traditional episodic memory tests for clinical and research applications.